# One Health surveillance approaches for melioidosis and glanders: The Malaysian perspective

**DOI:** 10.3389/fvets.2022.1056723

**Published:** 2022-12-15

**Authors:** Vanitha Mariappan, Kumutha Malar Vellasamy, Rohan Raaj Anpalagar, Yue-Min Lim, Nurhamimah Zainal Abidin, Sreeramanan Subramaniam, Sheila Nathan

**Affiliations:** ^1^Center for Toxicology and Health Risk Studies, Faculty of Health Sciences, Universiti Kebangsaan Malaysia, Kuala Lumpur, Malaysia; ^2^Department of Medical Microbiology, Faculty of Medicine, Universiti Malaya, Kuala Lumpur, Malaysia; ^3^Faculty of Applied Sciences, School of Biology, Universiti Teknologi MARA (UiTM), Cawangan Negeri Sembilan, Kuala Pilah, Negeri Sembilan, Malaysia; ^4^School of Biological Sciences, Universiti Sains Malaysia (USM), Georgetown, Penang, Malaysia; ^5^Chemical Centre Biology, Universiti Sains Malaysia (USM), Bayan Lepas, Penang, Malaysia; ^6^National Poison Centre, Universiti Sains Malaysia (USM), Georgetown, Penang, Malaysia; ^7^Faculty of Science and Technology, School of Biosciences and Biotechnology, Universiti Kebangsaan Malaysia, Bangi, Malaysia

**Keywords:** Malaysia, melioidosis, glanders, One Health, *Burkholderia pseudomallei*, *Burkholderia mallei*

## Abstract

The One Health concept was initiated to promote the integration of human, animal, and environmental ecosystems into healthcare to ensure effective control and the sustainable governance of multifaceted health matters. Climate change, deforestation, and rigorous farming disrupt the environment, which serves as the natural habitat for many animals and microbes, increasing the likelihood of disease transmission between humans and animals. Melioidosis (neglected tropical diseases) and glanders are of humans and animals caused by the gram-negative bacteria *Burkholderia pseudomallei* and its close relative *Burkholderia mallei*, respectively. In Malaysia, although melioidosis is endemic, it is not a notifiable disease. Hence, the true prevalence of melioidosis in Malaysia is unknown and varies in different regions of the country, with reported hotspots associated with agriculture-related activities. To date, no incidence of human glanders has been reported in Malaysia, although occupational exposure for equine handlers and veterinary professionals remains a concern. Additionally, antibiotics are widely used in the healthcare and veterinary sectors to treat or prevent *B. pseudomallei* and *B. mallei* infections, leading to the emergence of resistance in *B. pseudomallei*. Lack of surveillance, research, assessment, and management of glanders and melioidosis is a major issue in Malaysia. Proper assessment systems and cross-discipline cooperation are vital to recognize and manage both diseases. Experts and practitioners from clinical and veterinary disciplines, environmentalists, law enforcement, policymakers, researchers, local communities, and other experts need to communicate, collaborate, and coordinate activities to fill the knowledge gap on glanders and melioidosis to reduce morbidity and mortality rates in the country. This review aims to define the organizational and functional characteristics of One Health surveillance approaches for glanders and melioidosis from a Malaysian perspective.

## Introduction

Malaysia is a part of Southeast Asia and located north of the equator at latitude 4.2105°N and longitude 101.9758° and composed of two distinct non-contiguous regions: (i) Peninsular Malaysia, also called West Malaysia, which is on the Malay Peninsula (bordered by Thailand to the north and Singapore to the south) and (ii) East Malaysia, which is on the island of Borneo separated from Peninsular Malaysia by roughly 640 km of the South China Sea and share a land boundary with Indonesia (Kalimantan) on the south and Brunei is surrounded by a small coastal enclave. According to the World Bank, Malaysia is an upper-middle-income country and is recognized as one of the most rapidly developing countries in the world (1).

Malaysia is a tropical country with consistently high temperatures and humidity, heavy rainfall, and a climatic year patterned around the northeast and southwest monsoons. Due to its tropical weather, Malaysia is also an endemic hot spot for several types of infectious diseases, especially neglected tropical diseases. Both globally and in Malaysia, infectious diseases remain a major public health concern. Immense climate change and massive land development disrupt the habitat for many flora, fauna, and microbes, increasing the prospect of disease transmission between humans and animals. The Ministry of Health (MOH) Malaysia addresses pressures caused by infectious diseases by strengthening infectious disease surveillance, which enhances aptitude for early disease detection, swift repression of diseases, and the prevention of uncommon manifestations of infectious diseases. Observance and disease aptitude are essential in warranting rapid identification of emerging infectious diseases under the Prevention and Control of Infectious Diseases Act 1988 (Act 342) (2).

## One Health approach in Malaysia

Malaysian One Health strategies are unified, outlined, and formulated under the guidelines primarily of the World Health Organization (WHO) and other global organizations, including the Food and Agriculture Organization of the United Nations (FAO) and the World Organization for Animal Health (OIE). The main objective of these agencies is to promote collaborative efforts in response to food safety hazards, risks from zoonotic diseases, and other public health threats among humans, animals, and the environment, in addition to guiding efforts to reduce risks (3, 4). In general, the One Health concept was implemented to encourage holistic and trans-disciplinary approaches to achieve enhanced public health for humans, animals, and the environment (One Health Triads). The fundamental approaches used are communication, coordination, and collaboration (3Cs) among One Health Triads experts, including physicians, pharmacists, patients, epidemiologists, veterinarians, agricultural workers, environmentalists, ecologists, and wildlife experts to share their expertise in the One Health approach (5). The One Health concept that emphasizes the interdependence of human, animal, and environmental health has been adopted to promote multi-sector and trans-disciplinary collaboration at the local, regional, national, and global levels to achieve optimal health outcomes.

Malaysia has published action plans through a collaborative effort between the MOH and Ministry of Agriculture and Food Industry (MAFI) Malaysia to build a more holistic approach to combat threats related to (i) food safety and security, (ii) zoonotic diseases, (iii) AMR and difficult-to-treat bacterial infections, (iv) vector-borne diseases, (v) environmental protection from contaminants, and (vi) other health threats shared by the One Health Triads. In this action plan, other departments, including the Department of Veterinary Services (DVS) (animal aspects); Pharmaceutical Services Division (PSD) (environmental aspects); Medical Development Division, Institute for Medical Research (IMR), and the National Pharmaceutical Regulatory Agency (NPRA) (human health), Department of Fisheries (DOF) (aquaculture); Malaysia One Health University Network (MyOHUN) under the Ministry of Higher Education (MOHE) with National Health Education, Information and Communication Center (research, awareness and educational); Food Safety and Quality Division (food security), work together to achieve the goals (6).

## One Health: Melioidosis and glanders

On a national level, the Malaysia One Health University Network (MyOHUN) has sought to promote the One Health philosophy and spirit against zoonotic and infectious diseases by connecting and empowering government, universities, and allied organizations (7). Zoonotic diseases are defined as ‘diseases and infections which are naturally transmitted between vertebrate animals and man' (8). Zoonotic diseases pose a major threat to the One Health approach, with ~2.5 billion cases of human disease and 2.7 million fatalities worldwide annually resulting from newly emerging or re-emerging infectious diseases (9, 10). Surveillance and data exchange between the MOH and the DVS identified 14 priority diseases in Malaysia, such as highly pathogenic avian influenza, rabies, Rift Valley fever, anthrax, and other zoonotic diseases (11). Is it truly vital to identify zoonotic diseases and their etiology, their impact on society, and how to manage and control the diseases.

Melioidosis and glanders are currently not recognized as neglected diseases despite melioidosis having a significantly higher annual mortality rate compared to well-known diseases such as leptospirosis (54 vs. 5%) (12, 13). Approximately 165,000 human melioidosis infections are estimated to occur worldwide, with 89, 000 cases resulting in deaths annually (13). Melioidosis is prevalent in tropical and subtropical areas, particularly in Southeast Asian and Northern Australian regions. However, the infection can spread to other regions and melioidosis is most likely underreported in many countries (14). Unlike melioidosis, glanders, which mainly affect solipeds, have been eliminated in many developed countries through quarantine, euthanasia of infected animals, and the introduction of automotive vehicles (15). However, glanders cases continue to crop up regularly throughout Asia, Africa, South and Central America, and the Middle East.

Melioidosis and glanders are potentially fatal diseases caused by the gram-negative bacteria, *Burkholderia pseudomallei*, and its close relative, *Burkholderia mallei*. *B. pseudomallei* is a saprophyte found in its natural habitats, which are wet soil and water (including ponds, lakes, rivers, and seawater). However, *B. mallei* lacks a saprophytic reservoir and relies on an animal host to survive. Solipeds are the natural reservoir of *B. mallei* although it has been reported to be able to persist in water up to 30 days (16). Both bacteria are small, rod-shaped, aerobic, and non-spore-forming, with the exception that *B. pseudomallei* is motile while *B. mallei* is not. They are opportunistic intracellular pathogenic bacteria capable of infecting animals and humans. Glanders is recognized as a true zoonotic because of rare situations of zoonotic transmission to humans who come into contact with infected solipeds (17, 18). In contrast, *B. pseudomallei* is considered a sapro-zoonotic since it is an environmental saprophyte and zoonotic melioidosis is exceedingly rare (19).

Both pathogens commonly enter the human or soliped host by direct or indirect inoculation *via* skin abrasions or lacerations, inhalation of droplets or aerosolized bacteria, or through conjunctiva mucous membranes, oral, and nasal. Common manifestations of both diseases are fever, pneumonia, and abscesses on different organs. Antibiotics remain the mainstay of treatment for both melioidosis and glanders. Unfortunately, antibiotic treatment is increasingly becoming ineffective due to the development of antibiotic resistance by both pathogens, leading to a high risk of relapse and recrudescence. Currently, no vaccines or approved antibiotic prophylactics are available for either disease. In general, many major concerns related to these diseases are attributed to their diverse modes of transmission, resilience in survival, and AMR (20). These features have led to both *B. pseudomallei* and *B. mallei* being classified as Tier 1 select agents by the Centers for Disease Control and Prevention (21).

In Malaysia, melioidosis is endemic with over 1,000 cases reported in humans annually; nonetheless, it is still a non-notifiable disease unlike 25 diseases under Act 342 including dengue, tuberculosis, malaria, rabies and measles (20). Meanwhile, glanders has rarely been a public health concern, with only rare sporadic outbreaks reported globally (18). Hence, the full extent of both diseases in Malaysia remains largely unknown.

## One Health Triads: Melioidosis and glanders in Malaysia

Humans live in close proximity to wild and domestic animals and the environment. Undoubtedly, this close contact favors the spread of infectious diseases. In Malaysia, regions reported as hotspots for melioidosis are associated with agriculture-based activities, construction, and other soil/water-based activities. These activities involve frequent contact between humans and animals (livestock/farming), humans and the environment (farming/construction), and animals and the environment (livestock), highlighting a triad relationship in melioidosis (20). To date, no incidence of human glanders has been reported in Malaysia.

## Environmental contamination

According to a review of case reports of melioidosis in Malaysia, 39% of melioidosis cases were caused by some form of environmental exposure, highlighting the key role played by the environment in melioidosis incidence (22). As *B. pseudomallei* cells are readily found in the soil and water of endemic regions, the potential for environmental contamination by the bacteria is high (20).

Environmental contamination of *B. pseudomallei* can result from natural causes and human activity. Generally, melioidosis cases are the highest during the rainy and wet seasons (20, 23). Extreme weather and natural disasters such as floods and heavy rainfall have been associated with local outbreaks of melioidosis. Studies suggest that the surge in cases could have resulted from alterations to the superficial soil structure. Erosion of the surface soil layers and stagnated water in puddles or floods could expose the bacteria, typically found in the soil at depths of around 20–40 cm, and lead to proliferation on the soil surface. The presence of the bacteria on the surface increases the chances of direct contact as well as inhalation caused by the aerosolisation of the bacteria during rainy seasons, consequently increasing the risk of infections (24–26). Several cross-sectional studies conducted on post-flood soil samples in melioidosis hotspots across Malaysia have detected the presence of *B. pseudomallei* in superficial soil samples. A study conducted at Lubuk Yu, Pahang, after a melioidosis outbreak following a flood rescue mission, reported the presence of *B. pseudomallei* in a quarter of the surface soil samples and in 12% of soil samples obtained at a depth of 30 cm (24). Another study conducted in Kelantan detected *B. pseudomallei* in <1% of the samples (25). Both studies illustrate the influence of floods on the propagation of bacteria through the environment. The growth and spread of the bacteria due to natural phenomena could, ultimately, lead to *B. pseudomallei* contamination of the environment (23, 27).

Human activity also contributes to the environmental spread of *B. pseudomallei*. Activities such as agriculture and logging, which often involve clearing forests and tilling the land, disrupt the physicochemical properties of the soil (higher iron, water, and clay content). These changes could promote the dispersal of *B. pseudomallei* in the environment, leading to a higher risk of infection (28, 29). A case-control study to identify risk factors in farms for melioidosis during the monsoon season found that farms surrounded by bush-cleared or waterlogged areas recorded a higher number of animals in melioidosis cases (30). A study conducted in Kedah also found that melioidosis was more common in regions where the landscape has been greatly modified by human activity, such as commercial farming and cleared forests compared to intact forest regions, further supporting the impact of human activity on environmental contamination of *B. pseudomallei* (31). *B. mallei*, on the other hand, is unable to survive in soil or water, yet possesses the ability to aerosolise, hence, posing a risk to individuals in close contact with the bacteria. Reports on infections due to frequent exposure in laboratories working on *B. mallei* have been documented around the world (15, 18, 32). In short, exposure to both bacteria can be exacerbated by natural causes and human activity, which could lead to surges in infection and negatively affect the wellbeing of both animals and humans.

## Animal infections and spread

*B. pseudomallei* and *B. mallei* can infect various warm-blooded animals. *B. pseudomallei* can infect both wild and domestic animals ranging from house cats to gibbons while *B. mallei* primarily infect solipeds such as horses, donkeys and mules (18, 20). In Malaysia, melioidosis has been detected in orang-utans, sheep, goats, and other animals (20). A study conducted between 2012 and 2016 on the seropositivity of farm animals such as sheep, buffaloes, goats, and cattle, found that hotspots for livestock melioidosis were regions on the east coast of Peninsular Malaysia, namely Pahang, and the East Malaysian state of Sabah. Why these states recorded higher numbers of animal melioidosis is still unknown, although, factors such as rainfall patterns, physicochemical properties of the soil, waterlogging/flooding, bush clearing etc., could increase the contact of animals with *B. pseudomallei*, influencing the higher prevalence of animal melioidosis (28, 30, 33). However, due to lack of environmental data, the prevalence of melioidosis in hotspots areas was not able to be correlated with these factors. Overall, the prevalence of livestock melioidosis observed was under 1% in most regions, the highest recorded was in sheep, which was as high as 10% in Sabah (33). While the frequency of animal melioidosis is generally low, if not monitored closely, the possibility for major outbreaks remains. Such outbreaks could have devastating effects on the farmers' livelihood.

Another concern for zoonotic transmission stems from the global animal trade. The risk of imported animals carrying diseases to their destination countries is well-documented for melioidosis and glanders globally (34, 35). In Malaysia, the DVS has imposed regulations requiring equines to be glanders-free for up to 12 months pre-export. As for melioidosis, the DVS has no specific guidelines in place although it does require all imported animals to be free of infectious diseases and imposes compulsory quarantines for live animals upon arrival (36). These regulations reduce the risk of imported melioidosis, but without specific guidelines, the potential for imported animals to be infected remains a threat and could lead to epizootic outbreaks (34, 35).

The likelihood of melioidosis transmission from infected animals to humans is also significant, although rare. To date, only one confirmed case of zoonotic transmission of melioidosis from sheep to a human has been reported in Malaysia (15, 20). As for glanders, zoonotic transmission is the most common mode of transmission of glanders to humans. No zoonotic transmission of glanders has been reported in Malaysia, nonetheless, the risk of infection for individuals working closely with infected animals or contaminated animal material, is imminent (15, 18, 35).

## Human infections

To date, the number of melioidosis cases reported in Malaysia is thought to surpass 1,000 cases annually, however, under the Prevention and Control of Infectious Diseases Act 1988, no provision is made to establish melioidosis as a notifiable disease at a national level (20). The current guidelines for clinical and public health management of melioidosis in Malaysia are only available at the state level albeit in only limited states such as Pahang and Sabah, developed by their respective State Health Departments. These guidelines include regulations for diagnosis, treatment regiments, soil sampling in endemic regions, preventive and control measures in endemic regions as well as notification to the state health authorities through the establishment of melioidosis registries (37). The success of the introduction of the Pahang Melioidosis Registry was captured by a retrospective study conducted a year after the introduction of the registry where a decrease in mortality by 10% (54–44%) in adult melioidosis deaths and culture-confirmed melioidosis deaths, from 19 to 0% (38). This retrospective study highlights the vital role played by notification in the prevention and control of infectious diseases.

The distribution of cases in Sarawak and Pahang can be attributed to the widespread agrarian socioeconomic activities in the region which often involve contact with soil and water, as aforementioned (39, 40). A study conducted in the northern region of Malaysia (Alor Setar, Kedah) found that individuals participating in fishing, farming, and forestry were significantly more prone to acquire melioidosis solidifying the former's claims (40). In another study, Deris et al. (41) reviewed 35 melioidosis case reports from 2001 to 2005, at the Hospital Universiti Sains Malaysia, Kubang Kerian, Kelantan (northern Malaysia). Following that, Zueter et al. (23) performed a retrospective analysis of 158 confirmed cases of melioidosis collected from medical records from 2001 to 2015. It was carried out to update the current status of melioidosis clinical epidemiology in high-risk regions of the country where exposure to environmental elements was recorded during patients' activity prior to admission, including farming, military work or recent travel to the jungle or swimming in natural ponds and any other work. In short, occupational risks play a key role in propagating melioidosis among humans. As for urban regions such as Kuala Lumpur, however, little is known about the epidemiology of urban melioidosis in Malaysia hence more research is required.

Human infections of melioidosis in Malaysia, account for mortality rates within the range of 35–54%. The high mortality is often attributed to lack of awareness and inefficient /slow diagnosis and treatment (20). In clinical settings, melioidosis frequently presents as pneumonia (around 40%), attributed to the possibility of inhaled infections, followed by soft tissue infections (22, 39, 42). Due to the pathogen's opportunistic nature, several predisposing factors prevalent in melioidosis patients have also been identified. The major two factors are diabetes, prevalent in over 75% of cases, and renal failure (10%) (20). In Malaysia, a routine blood culture test (Francis media agar, MacConkey agar, blood agar, and chocolate agar) is carried out as part of the sepsis diagnosis for patients with fever. Confirmation of *B. pseudomallei* is done either by API 20NE biochemical kit or automated biochemical systems (Vitek 2 or MALDI-TOF MS). Antibiotic susceptibility tests and *B. pseudomallei*-specific latex agglutination assay are recommended tests but are not widely used due to financial limitations. Serological tests to detect the presence of anti-*B. pseudomallei* antibody titres using indirect hemagglutination assay (IHA) or enzyme-linked immunosorbent assay (ELISA) are widely accepted as unreliable for diagnosing melioidosis in Malaysia (20). Although *B. mallei* is not diagnosed routinely in Malaysia, it can be determined by blood culture, biochemical tests, and automated biochemical systems.

Understanding the full burden and epidemiology of melioidosis in humans in Malaysia calls for larger-scale studies as currently, most studies conducted only cover small populations and limited regions or are dated which may not highlight the prevalence of melioidosis in Malaysia accurately.

## Melioidosis and glanders surveillance in Malaysia

Surveillance of an infectious disease is a crucial factor in monitoring and managing the health of a population as it provides means to understand the existing burden and epidemiology of the disease, monitor the trends of the disease, and also detect outbreaks and spread of new strains (43). Malaysia, despite being among the hotspots for melioidosis in the Southeast Asian region, does not have an effective surveillance system for these diseases. This presents a huge gap in the big picture of disease burden, clinical presentations, and diagnosis and treatment of the disease in Malaysia (20). Different local research groups have conducted various surveillance studies including serological, clinical, and epidemiological surveillance. However, these reports are sporadic and proper surveillance by the relevant government bodies is still lacking.

AMR is a major threat emerging across various bacterial diseases and infections. Over the years, studies have also indicated an increase in the development of AMR among the *B. pseudomallei* isolates in Malaysia, as the primary form of treatment is still antimicrobial therapy due to the lack of vaccines (44). Both bacteria have adapted a variety of mechanisms to successfully evade antimicrobial action. For example, both *B. pseudomallei* and *B. mallei* are intrinsically resistant to β-lactams while *B. pseudomallei* is also resistant to aminoglycosides (45). In addition to the intrinsic resistances, the variable resistances between different strains of *B. pseudomallei*, could also diminish the effect of the antimicrobial therapy, failing to clear the infection, increasing the likelihood of relapses or mortality (29, 44, 46, 47). Antibiotic therapy for glanders, on the other hand, is largely understudied, hence, the effectiveness of antibiotic therapy in humans infected with glanders is unknown (44, 46).

In melioidosis, reports on resistance toward antimicrobial treatments are relatively common. Khosravi and colleagues reported the detection of six multidrug resistance (MDR) isolates demonstrating resistance to meropenem, imipenem and ceftazidime among the 81 clinical *B. pseudomallei* isolates tested. Carbapenems, a potent class of antimicrobials, are often used in treating severe or septicaemia infections of *B. pseudomallei* while ceftazidime, a cephalosporin is a common antibiotic used in the initial phases of melioidosis treatment (47, 48). The rest of the isolates demonstrated resistance to at least one of the antibiotics tested including chloramphenicol, amoxicillin/clavulanic acid, doxycycline, tigecycline, clarithromycin, and trimethoprim/sulfamethoxazole which are used in various treatment regiments such as eradication therapy and second-line therapy (47, 48). The emergence of resistance toward antimicrobials used in melioidosis treatment could lead to complications in the established treatment protocols for melioidosis by failing to clear the infection successfully, increasing the likelihood of relapses or mortality, or necessitating the administration of different or more potent antimicrobials or in severe cases, the failure of antimicrobial treatment (29, 44, 46, 47). Differences in AMR between strains could impede local efforts in establishing a uniform and effective treatment scheme for melioidosis (47, 49). On the other hand, antibiotic therapy for glanders in Malaysia is largely understudied, hence, the effectiveness of antibiotic therapy in humans infected with glanders is unknown (44). However, several researchers have reported on different antibiotic susceptibility profiles of *B. mallei*, which appear to resemble *B. pseudomallei* since they are similar in terms of biochemical, antigenic, and pathogenicity properties (50). According to Heine et al. (51), the *in vitro* susceptibilities of 11 *B. mallei* strains against 28 antibiotics demonstrated susceptibility to aminoglycosides, macrolides, quinolones, doxycycline, piperacillin, ceftazidime, and imipenem. Following that, Thibault et al. (52) tested 15 *B. mallei* for their susceptibilities to 35 antimicrobial agents (antimicrobial agents that have not been tested before) and obtained lower susceptibility against fluoroquinolones and aminoglycosides. In contrast, Naureen et al. (53) determined that *B. mallei* isolated from natural outbreaks of equine glanders were intrinsically resistant to ampicillin and 17% of isolates were resistant to amoxicillin, cephradine, cefuroxime, ceftizoxime, ceftriaxone and norfloxacin. However, none of the isolates was resistant to amoxicillin-clavulanic acid, doxycycline, chloramphenicol, gentamicin or trimethoprim-sulphadiazine. Overall, the susceptible patterns of *B. mallei* revealed that the susceptibilities measured are dependable with the commendations for the treatment of *B. mallei* infections.

Ahmad et al. (54) demonstrated high susceptibility of clinical isolates to the antibiotics that were used for initial treatment and multiple resistances toward antibiotics routinely used for maintenance therapy including chloramphenicol, tigecycline, and ciprofloxacin. Subsequent studies have reported the emergence of resistance toward both the initial and eradication phase antibiotics as well as the development of MDR among Malaysian isolates. A study in Kedah, one of Malaysia's hotspot regions for melioidosis, also reported 53–94% resistance to trimethoprim/sulfamethoxazole, gentamicin, and amikacin among the 228 isolates tested (55). This may pose a serious clinical implication in terms of patient management if the therapeutic efficacy of antibiotics currently used for treating melioidosis is compromised. Thus, we recommend that MOH should consider the incorporation of AMR surveillance for *B. pseudomallei* into the NSAR (www.imr.gov.my/MyOHAR/) programme to ensure proper management of melioidosis in Malaysia. To this point, the *B. pseudomallei* AMR pattern has been observed and stated in the NPRA report from 2005 to 2012 (2004–2013, MOH) (Table 1). No further report has been produced thus far.

**Table 1:** *Burkholderia pseudomallei* antibiotic resistance (2005–2012) (National Pharmaceutical Regulatory Agency report 2004–2013, MOH; https://www.imr.gov.my/MyOHAR/index.php/site/archive_rpt).

**Antibiotics**	**Year/percentage (number of isolates)**							
	**2005**	**2006**	**2007**	**2008**	**2009**	**2010**	**2011**	**2012**
Amikacin	78 (123)	NA	88 (325)	85.8 (212)	81.2 (128)	80.3 (238)	79.9 (329)	79.1 (258)
Ampicillin	NA	NA	98.5 (85)	98 (50)	100 (23)	82.6 (23)	91.1 (56)	97.3 (73)
Amoxicillin/Clavulanate	5.5 (164)	NA	5.9 (220)	8.2 (281)	26.4 (174)	18.5 (276)	21.2 (539)	22.8 (451)
Ceftazidime	4.4 (181)	NA	2.8 (358)	0.6 (344)	0.4 (245)	1.7 (349)	1.9 (618)	3.5 (566)
Cefotaxime	NA	NA	4.2 (72)	8.3 (48)	8.1 (37)	0 (46)	8.8 (68)	14.7 (75)
Cefepime	5.6 (36)	NA	8.2 (159)	8.8 (125)	5.3 (38)	4.2 (95)	13.3 (195)	9.7 (73)
Gentamicin	95.3 (129)	NA	97.1 (313)	96.8 (190)	NA	NA	NA	NA
Imipenem	0.7 (134)	NA	0.3 (371)	1.8 (342)	1.2 (244)	2.3 (345)	0.6 (620)	2.4 (550)
Meropenem	NA	NA	2.8 (212)	2.7 (200)	7.7 (247)	4.1 (292)	11 (554)	6.2 (483)
Netilmicin	87.9 91)	NA	90.3 (134)	87.5 (104)	NA	NA	NA	NA
Ampicillin/Sulbactam	1.8 (55)	NA	2.9 (137)	5.1 (98)	5.6 (54)	5.8 (69)	5.7 (192)	1.4 (141)
Piperacillin/Tazobactam	3.6 (28)	NA	0.9 (215)	0.7 (143)	0 (75)	1.9 (105)	2 (255)	0.7 (142)
Trimethoprim S	NA	NA	45 (269)	53.2 (293)	46.9 (211)	40.1 (329)	44.2 (520)	38.2 (437)

NA, data not available.

The CLSI Performing Standards for Antimicrobial Susceptibility testing guidelines. When the interpretation zones were not available in the CLSI, the EUCAST guidelines were referred to.

Meanwhile, in northern Australia, the Darwin Melioidosis Guideline 2015 describes a new treatment paradigm where the antibiotic duration of intravenous therapy (minimum of 2 weeks) is determined by the site and severity of melioidosis. Similar to therapy in Malaysia, ceftazidime is used for most cases and meropenem is kept for those with severe melioidosis. Oral intake of dual therapy, trimethoprim-sulfamethoxazole, is given during intensive phase therapy for better tissue penetration and to limit the emergence of resistance. However, in contrast, dual therapy was not shown to improve mortality in patients with severe melioidosis in Thailand (56, 57).

The history of nationwide sero-surveillance studies involving melioidosis dates back to the 1960s, conducted by the United States of America Medical Research Unit (USAMRU) in various states in Malaysia (20). Despite the early start, continuous sero-surveillance is still lacking in comparison to other countries in the region, leading to the failure of creating well-established data associated with melioidosis in Malaysia. A seroprevalence study using a hemagglutination-based assay found the highest prevalence of antibodies against *B. pseudomallei* in the states of Kedah and Sabah. The high prevalence in these states correlated with high-intensity rice farming, which increases the exposure to contaminated soil (58). Many recent studies have also supported this as higher seropositive cases were reported in the Malaysian east coast states of Kelantan, Terengganu, and Pahang as well as Sarawak where there is a predominance of environmental-related economic activities involving agriculture, farming, fishing, and forestry (59, 60).

More recently, a study by Hadi and colleagues from the IMR Malaysia reported on the melioidosis seropositivity of patients admitted to healthcare centers in Malaysia from 2015 to 2019 where they found that the territory of Sarawak (15.1%) recorded the highest number of seropositive cases followed by the state of Pahang (13.9%) and Kuala Lumpur (11.3%) (61). Overall, from more than 26,000 sera samples tested for IgM against *B. pseudomallei*, ~16.4% seropositivity was recorded with the majority of the seropositive patients aged over 55 years old. The age group below 15 years old was found to be the most susceptible to melioidosis infection. There was an increasing pattern of seropositive cases from 2015 to 2017 (2.0–6.4%) followed by a decrease to 2.3 and 1.2% in 2018 and 2019, respectively. Increased awareness of melioidosis in recent years may have contributed to the reduced seropositive cases, especially in some states like Pahang and Sarawak where melioidosis has been made a notifiable disease (60).

Although scarce, seroprevalence studies of melioidosis in animals have also been reported. Based on the data collected from the DVS and the Veterinary Research Institute (VRI) from the year 2000–2009, a varying seroprevalence of melioidosis was reported in the different livestock 7.6% (cattle), 48.2% (buffalos), 2.6% (goats), 13.6% (sheep), and 3.6% (pigs). The overall yearly seroprevalence of melioidosis in this livestock was reported to vary throughout the 10 years with an average of 6.9% (62). The species-specific seropositivity was found to be reduced in a seroprevalence study between the year 2012–2016 conducted with the data from VRI, reported as 0.7% (cattle), 0.99% (buffalos), 0.42% (goats), and 0.9%(sheep) with an average yearly prevalence of 0.75% (33), In 2015, a study by Hambali and colleagues have reported a very low seroprevalence of melioidosis in the sheep and goats (1 and 0%, respectively) from a selected farm (63). Although there is a marked reduction in the seroprevalence in animal reported over the years, this needs to be treated with caution as the number of animals included in some of the categories were very small compared to the others. Furthermore, farm management practices such as intensive or semi-intensive farming systems may also affect the seroprevalence reported. Farm animals that are intensively raised may have less contact with the soil as food and water will be provided to them in their containment facility. In contrast, semi-intensively raised animals are allowed to graze in the fields for a period of time every day before they are caged in their containment (64). Hence, semi-intensively raised farm animals are expected to have a higher seroprevalence of melioidosis as they are more exposed to contact with the soil and water during grazing (62). Thus, a more structured sero-surveillance of melioidosis in animals is warranted, not only including the DVS and VRI but also the Department of Environment (DOE) under the Ministry of Environment and Water, Malaysia. This is crucial as various factors including the geographical regions, environmental factors including the difference in temperature, weather, rainfall intensity, type of soil, and other factors that influence the survival of *B. pseudomallei* in the soil and water need to be taken into consideration in conducting this sero-surveillance to obtain a meaningful data set.

In the case of glanders, despite the lack of surveillance data, DVS has imposed various regulations including requirements that all imported animals be free of infectious diseases and imposes compulsory quarantines for live animals upon arrival (36). Glanders has also been included under the animal-borne disease list of the National Action Plan on Invasive Alien Species (NAP IAS) 2021–2025. This NAP IAS from the Department of Agriculture (DOA) Malaysia aims to utilize an integrated approach of research, education, and awareness coupled with increased inspection and control at entry points and international borders in order to control the establishment and spread of IAS (3, 65). Additionally, glanders has been included in the list of 121 diseases that are compulsory to be notified authorities under Section 31 (1) Animals Act 1953 (Act 647) (66). Under this section, it is the responsibility of all owners or animal custodians and veterinarians to report any incidence of the listed diseases to the nearest veterinary authorities, whether suspected or confirmed. Collectively, these efforts will allow control of the disease as glanders can be transmitted to humans by direct contact with diseased animals or with infected or contaminated material, with occupational exposure continues to remain as serious concern and a key risk factor for equine handlers, veterinary professionals and households exposed to equids with glanders.

In Malaysia, the lack of surveillance, research, assessment, and management of glanders and melioidosis infections is a major issue. Proper assessment systems and cross-discipline cooperation are vital to recognizing and managing these diseases. The One Health approach advocates the involvement of experts from healthcare, animal, environmental and other relevant sectors to monitor and control public health threats and to understand how diseases spread among different ecosystems. To ensure effective public health interventions, it is critical that human, animal and environmental health partners collaborate to successfully control, prevent or eradicate zoonotic diseases, improve food safety and security, reduce AMR infections and protect global health security. Disease surveillance requires early detection and diagnostic testing of suspected clinical cases. Fast and effective diagnostics are required for the clinical diagnosis of melioidosis. In Malaysian laboratories, the isolation and identification of *B. pseudomallei* are dependent on sample culture on selective agar, followed by biochemical tests that can take up to 5 days. Unfortunately, many patients afflicted with acute melioidosis could die before the results are available (20). Although molecular-based identification of *B. pseudomallei* is currently available in local laboratories and has the advantage of a shorter diagnostic turnaround time, the sensitivity of the tests could be affected by the presence of inhibitors (67). Therefore, bacterial culture remains the gold standard for diagnosis.

## Suggestions to enhance policies, enforcement, and surveillance in Malaysia

Awareness of melioidosis across Malaysia is still lacking among healthcare personnel and the public. It is well recognized that although the burden of melioidosis is primarily on human health, disease control and surveillance need to be also focused on livestock and wildlife as animal melioidosis has been reported in local animals (29, 63, 68, 69). The species of animals affected by melioidosis and the diverse locations from which animal cases have been reported indicate a wide distribution of *B. pseudomallei* in the environment in Malaysia (20). The zoonotic potential of glanders and the prospective threat of *B. mallei* as a biological weapon (70, 71) makes glanders a disease of public and biodefense concerns. As both *B. pseudomallei* and *B. mallei* are highly infectious with high mortality and limited treatment options, there is an urgent need for active surveillance of the diseases to control the emergence of an outbreak in Malaysia.

As glanders and melioidosis are a One Health issue, implementing protocols toward controlling the disease threats requires enhanced awareness across multi-sectorial groups and collaboration between the environmental, animal, and human health sectors. A successful communication strategy is crucial to raise awareness but also to educate the public to prevent and control melioidosis and glanders in the community. Heightened awareness among healthcare personnel is essential for early diagnosis followed by prompt appropriate treatment. It is worth highlighting that pediatric melioidosis is of concern in Malaysia and awareness among pediatricians is crucial to avoid a delay in diagnosis and treatment in children (20, 60). Meanwhile, those at risk of occupational exposure such as paddy farmers, soldiers, and laboratory workers should be made aware of the disease to protect them from being infected with the pathogen. In the case of glanders, although zoonotic *B. mallei* in humans appear uncommon, cases have been reported infections due to close contact with infected animals or with *B. mallei* cultures in the laboratory (18, 71, 72). Therefore, awareness needs to be extended to those who are at higher risk to handle the pathogen namely veterinarians, farmers, horse traders, and personnel working in stables and slaughterhouses. Those with occupational exposure are advised to have yearly medical check-ups. If there is a melioidosis or glanders outbreak, targeted surveillance of the human population is recommended. It could be suggested that the MOH, DVS, and other relevant stakeholders in One Health develop a National Strategic Plan for melioidosis and glanders as an initiative to build national awareness and surveillance of the diseases.

Well-planned control programs and campaigns along with commitment, strong political support, and adequate resources provided by the government are key to the success of controlling both diseases. In Malaysia, such a strategic plan has shown good progress toward the elimination of several infectious diseases such as malaria (73) and lymphatic filariasis (74). There have also been suggestions to initiate an annual celebration of “Melioidosis and Glanders Day” which could create national or global awareness of both melioidosis as well as glanders. People can be reminded about the importance of these diseases, and this could indirectly strengthen closer intersectorial collaboration. The far-reaching networks of these multi-sectorial agencies are the glue that holds the integrated strategy together, underpinning the One Health framework.

Investments in education and training are essential for the capacity building of a trained workforce for disease surveillance. More microbiology laboratories need to be equipped with standard diagnostic testing facilities, especially in rural areas, where the probability of a *B. pseudomallei* infection is anticipated to be high. Once the endemic areas are identified from the sero-surveillance studies, isolation, and identification of *B. pseudomallei* from the environment are essential to assist in determining geographical areas at risk and revisiting the risk assessment of melioidosis. In addition, a robust system for reporting the diseases should be in place to assess national endemicity and disease burden. Data sharing and excellent communication between diagnostic laboratories and field staff working in human health, animal health, and the environment are key to the One Health approach to control these diseases. Similar strategies can be implemented for the management and control of glanders, which include early detection, and diagnostic testing of suspected clinical cases, routine screening of asymptomatic equids, and culling with proper disposal of infected animals. Collectively, these will enhance the recognition of the diseases in humans, animals, and the environment. A good understanding of the epidemiology of melioidosis and glanders in the country will assist in establishing a targeted and effective surveillance system that could support priority setting for neglected tropical diseases by policymakers and ensure adequate funding for adopting and maintaining these policies.

There is a need to leverage funding resources both from local and international agencies to support research on glanders. Knowledge, attitude, and practice (KAP) surveys and socio-cultural and economic studies are essential to generate data that would assist in reinforcing current practices and better inform future strategies and policies. More studies are required to unravel the complexity of *B. pseudomallei* and *B. mallei* and identify protective antigens that pave the way for the development of effective novel vaccines to protect against acute and chronic infections. In addition, law enforcement and policy formulation add another layer to the surveillance strategy. Preparedness plans should be in place to quickly deal with potential glanders or melioidosis outbreak to contain the disease. An example is the implementation of an international veterinary certificate that requires horses to be free from equine diseases including glanders for 6 months before export, with no clinical signs of any infectious disease at the time of export, and to be quarantined upon arrival (75). In the event of a glanders or melioidosis outbreak, the relevant authorities should investigate how individuals were exposed to the pathogens. It is also urgent to identify other individuals who may have been exposed so they can begin post-exposure prophylaxis or treatment. Multi-sectorial efforts are essential to push forward the legislation for melioidosis and glanders to be declared as notifiable diseases.

## Conclusion and future perspective

Unlike dengue and tuberculosis, melioidosis is still not considered a notifiable disease in all states in Malaysia. One limitation in implementing the One Health approach in Malaysia is the separation of local health, agriculture, and environment government authorities, with each agency claiming ownership of regulations that relate to One Health. Thus, all relevant stakeholders should work together in a concerted effort to ensure the collective goal of One Health to attain optimal health for people, animals, and the environment (Table 2). As has been attempted globally, Malaysia should undertake to reinforce and strengthen collaboration across various disciplines to ensure One Health surveillance is executed at all levels to control potential threats to humans, animals, and environmental ecosystems. A Malaysian Melioidosis/Glanders Network (MMN) should be initiated to integrate across human, animal, and environmental health, fundamental and clinical research, biosafety and biosecurity and economic impacts. This network should be able to connect and unite researchers and health implementers throughout the country to improve understanding of the epidemiology, surveillance, diagnosis, and treatment of this highly endemic and under-diagnosed disease. Lessons could be also learned from our neighboring country, Thailand, where melioidosis is endemic and the mortality rate is much higher. Awareness of melioidosis and glanders should also be created and emphasized amongst the medical profession and public through population-based-survey and public engagement campaigns utilizing video clips, as potential educational tools.

**Table 2:** Proposed One Health implementation activities on melioidosis and glanders diseases.

**Program/Department/Organization**	**Objectives**	**Suggested activities/policies**
**Human**		
Ministry of Health (MOH) Institute of Medical Research (IMR) State Health Departments Ministry of Higher Education (MOHE)	• To ensure proper management of melioidosis in Malaysia • To ensure effective public health interventions to eradicate melioidosis • To ensure appropriate use of antibiotics and control development of AMR in *B. pseudomallei* • To ensure the availability of a trained workforce for treatment and diagnosis of melioidosis	• Structured nationwide surveillance of human melioidosis • Changes in policies to include melioidosis as a notifiable disease in all states • Include melioidosis in the NSAR programs • Investments in education and training • Continuous research and development • Perform epidemiological studies • Knowledge, attitude, and practice surveys
**Animal**		
Department of Veterinary Services, Malaysia (DVS) Veterinary Research Institute (VRI)	• To identify the prevalence of melioidosis in animal and farm animal • To prevent and control infections	• Structured nationwide surveillance of melioidosis in animal • Perform epidemiological studies • Knowledge, attitude, and practice surveys • Control the importation of animals
**Environment**		
Department of Environment (DOE) Ministry of Environment and Water, Malaysia.	• To determine geographical areas at risk • To determine prevalence of *B. pseudomallei* in the soil	• Determine the prevalence of *B. pseudomallei* in soil and water in the different geographical regions
**Human, animal, and environment (One Health Triads)**	
Ministry of Health (MOH) Department of Veterinary Services, Malaysia (DVS) Veterinary Research Institute (VRI) Malaysia One Health University Network (MyOHUN) Department of Environment (DOE)	• To monitor, manage and control public health threats of melioidosis • To understand the spread of melioidosis among different ecosystems • To provide training to the personnel staff/those handling animals/working closely with soil/water • To enhance research/rapid diagnostic/vaccine/	• Build national awareness of the diseases • Provide personal protective equipment to workers • Integrated effort to control the development of AMR in *B. pseudomallei*

## Author contributions

VM and KV: conceptualized the manuscript. VM, KV, RA, Y-ML, and NZ: wrote the manuscript. VM, KV, SS, and SN: revised and edited the manuscript. All authors have critically reviewed the manuscript.
